# A novel predictive strategy for the incidence of postoperative neurocognitive dysfunction in elderly patients with mild cognitive impairment

**DOI:** 10.3389/fnagi.2022.985406

**Published:** 2022-09-29

**Authors:** Yueying Liang, Xi Xin, Hongyan Wang, Wei Hua, Yi Wu, Xinyi Wang, Ping Li, Tong Zhou, Haiyun Wang

**Affiliations:** ^1^Department of Anesthesiology, The Third Central Hospital of Tianjin, Tianjin Key Laboratory of Extracorporeal Life Support for Critical Diseases, Artificial Cell Engineering Technology Research Center, Tianjin Institute of Hepatobiliary Disease, Nankai University Affinity The Third Central Hospital, The Third Central Clinical College of Tianjin Medical University, Tianjin, China; ^2^Department of Anesthesiology, Tianjin Hospital, Tianjin, China

**Keywords:** elderly, mild cognitive impairment, postoperative neurocognitive dysfunction, cerebral oxygen saturation, plasma amyloid β-42

## Abstract

**Objective:** Preoperative levels of cognition-related biomarkers and intraoperative cerebral ischemia and hypoxia might cause postoperative neurocognitive dysfunction (PND). The aim of this study was to evaluate the predictive ability of preoperative plasma biomarkers along with cerebral oxygen saturation (SctO_2_) for the incidence of PND in elderly patients with mild cognitive impairment (MCI).

**Methods:** A total of 210 patients aged 65–80 years undergoing spinal surgery were randomly assigned to three groups (*n* = 70 each): propofol, sevoflurane, and propofol/sevoflurane as anesthesia maintenance protocols. Propofol was administrated target-controlled infusion of 4 μg/ml (group P), the minimum alveolar concentration (MAC) of inhalation anesthetic sevoflurane was 1.3 (group S), and propofol was injected with a target-controlled plasma concentration of 1.2 μg/ml, accompanied by sevoflurane inhalation 0.7 MAC (group PS). Cognitive function was evaluated 1 day preoperatively and on the 7th day postoperatively. Preoperative levels of amyloidβ-40 (Aβ-40), Aβ-42, total tau protein (T-tau), phosphorylated tau protein (P-tau), and triggering receptors on myeloid cells-2 (TREM2) were investigated. SctO_2_ was monitored intraoperatively.

**Results:** Aβ-42 had the strongest significant correlation with preoperative MoCA score. The value of Aβ-42 associated with a high risk of PND was 28.34 pg/ml, and the area under the curve (AUC) was predicted to be 0.711. When the preoperative level of Aβ-42 was 28.34 pg/ml, SctO_2max_% was 9.92%. The AUC was predicted to be 0.872, and the sensitivity and specificity were 0.833 and 0.841, respectively.

**Conclusion:** Under the conditions of preoperative Aβ-42 less than 28.34 pg/ml, the intraoperative fluctuation range of cerebral oxygen saturation should be maintained within 9.92% to reduce the occurrence of PND in geriatric patients with MCI.

## Introduction

Postoperative neurocognitive dysfunction (PND) is an objectively measured decline in cognition compared with that of the preoperative state (Evered and Silbert, 2018) and it adversely affects the quality of life and rehabilitation of patients (Lin et al., 2020). The clinical diagnosis of PND is a long process that requires the application of various cognitive tests and postoperative follow-up (Miniksar et al., 2021). Preoperative cognitive function is an important factor that cannot be ignored. PND leads to neurological complications due to multifactorial causes, with a high rate of morbidity, especially in geriatric patients with mild cognitive impairment (MCI; Bekker et al., 2010; Kline et al., 2012; Xin et al., 2021). A retrospective analysis of 2014 patients aged above 65 years who underwent surgery found that the incidence of postoperative delirium in patients with MCI before surgery was significantly higher than in patients of the same age with normal cognitive function before surgery (8.7% vs. 2.6%; Sprung et al., 2017). Preoperative plasma biomarkers are closely correlated with MCI and subsequent cognitive dysfunction. Identification of the most optimal-related biomarkers and their proper concentration could enhance PND prevention and treatment practices.

MCI is defined as the clinical stage between the expected cognitive decline of normal healthy aging and a more serious decline characterizing dementia (Ng et al., 2021). The incidence of MCI is 10%–20% of adults above 65 years of age, and approximately 20%–40% of patients with MCI progress to dementia every year (Skolariki et al., 2021). As the population ages, surgery is being performed more frequently, and in progressively older adults with MCI. The inappropriate perioperative management of patients with MCI might increase the incidence of PND and accelerate the progression of MCI to Alzheimer’s disease (AD; Racine et al., 2018; Xin et al., 2021). So the choice of appropriate anesthetic drugs is important to reduce the incidence of PND in patients with MCI (Xin et al., 2021). We previously conducted preliminary research on the types, dosages, and compatibility of general anesthetics for patients with MCI and confirmed that the combination of 0.7 minimum alveolar concentration (MAC) sevoflurane with 1.2 μg/ml propofol (plasma target concentration) is safe for MCI patients undergoing surgery with general anesthesia. Closely related biomarkers contributed uniquely to predict the occurrence of PND.

Intraoperative cerebral ischemia and cerebral oxygen desaturation have been proposed as possible mechanisms of PND (Lei et al., 2007). Optimizing cerebral perfusion may allow for reducing the risk of PND. Cerebral oxygen saturation (SctO_2_) based on near-infrared spectroscopy monitoring depends on the balance of cerebral oxygen supply and consumption. During noncardiac surgery, a well-maintained level of SctO_2_ can help reduce the incidence of intraoperative cerebral ischemia in elderly patients (Casati et al., 2005). Studies have shown that the change in intraoperative SctO_2_ has important implications in PND (Ni et al., 2015). Intraoperative SctO_2_ less than 50% or a greater than 20% decline from baseline is an independent risk factor for PND (Kim et al., 2016). However, the available literature regarding the relationship between SctO_2_ and PND in elderly patients with MCI undergoing major surgeries is minimal. Further, whether the combined assessment of preoperative plasma biomarkers and SctO_2_ is also effective to clinically reduce the incidence of PND is still unclear.

Given these limitations, we hypothesized that screening biomarkers closely correlated to MCI, and limiting the fluctuation in SctO_2_ based on near-infrared spectroscopy monitoring might further prevent PND in elderly patients with MCI. Hence, the present study was designed to elucidate the predictive capability of preoperative biomarkers and SctO_2_ for PND in elderly patients with MCI undergoing surgery.

## Methods

### Study design

The randomized clinical study was conducted with the approval of the Institutional Human Research Ethics Committee of the Third Central Clinical College of Tianjin Medical University (IRB2019-011-01) and is in compliance with the Helsinki Declaration. The study was registered at the Chinese Clinical Trials Registry Center before patient enrollment (Registration No.: ChiCTR2000038307). Written informed consent for participation in this study was obtained from all patients.

### Participants

**Figure 1:**
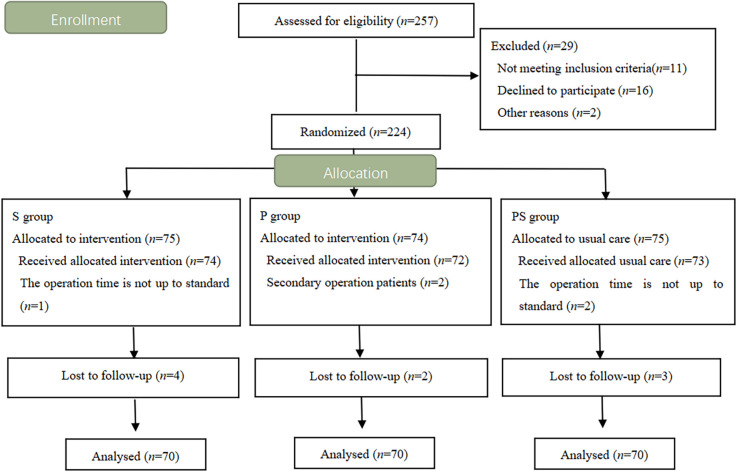
Flow chart of patients included in the study.

Patients aged 65–80 years and scheduled for spinal surgery were enrolled between June 2019 and July 2020. Sex, body mass index (BMI), and American Society of Anesthesiologists (ASA) physical status II or III were recorded. MCI was diagnosed clinically based on the following criteria (Radtke et al., 2013): subjective memory loss stated by self or family members preoperatively, Montreal Cognitive Assessment (MoCA) score in the range of 15–24, mini-mental state scale (MMSE) score less than 27 points (it depends on the level of education), Clinical Dementia Rating (CDR) of 0.5 points, and activities of daily living (ADL) score less than 26 points. Patients were excluded from the study if they met any of the following criteria: preoperative neurological diseases (such as vascular dementia), severe liver and renal insufficiency, autoimmune diseases, recent use of sedatives, antidepressants, or immunosuppressive drugs, traumatic brain injury or history of alcoholism, and previous participation in the study. In all, 224 patients were enrolled and randomly divided into three groups (*n* = 70): propofol group (group P), sevoflurane group (group S), and propofol/sevoflurane group (group PS; Figure 1).

### Anesthetic management

Heart rate (HR), blood oxygen saturation (SpO_2_), invasive arterial pressure, electrocardiogram (ECG), bispectral index (BIS), and SctO_2_ were continuously monitored in all patients during the perioperative period. Two sensors of a cerebral oximeter were placed on the left and right sides of the forehead for continuous SctO_2_ monitoring until the end of administration of anesthesia. When SctO_2_ is a greater than 20% oxygen saturation reduction from baseline or an absolute value of less than 50%, the patient’s head position was checked, and the mean arterial pressure (MAP) was optimized. Surgeons and anesthesiologists were blinded to the patients’ group allocations and the measurement of SctO_2_ to exclude subjective bias.

All patients received intravenous anesthesia following the same induction protocol with 0.05–0.2 mg/kg midazolam, 0.3–0.6 μg/kg sufentanil, 0.3 mg/kg etomidate, and 0.2 mg/kg cisatracurium. Mechanical ventilation was initiated after endotracheal intubation, and the ventilator parameters were adjusted: inhaled oxygen concentration 60%, oxygen flow rate 1 L/min, respiratory rate 12–14 /min, tidal volume 8–10 mL/kg, inhalation and exhalation ratio 1:2, and maintenance of partial expiratory carbon dioxide pressure between 35 and 45 mmHg (1 mmHg = 0.133 kPa). Group P was administrated target-controlled infusion of propofol, and the plasma target-controlled concentration was 4 μg/ml. Group S was administered 1.3 MAC of sevoflurane by inhalation. In the PS group, propofol was injected with a target-controlled plasma concentration of 1.2 μg/ml, accompanied by sevoflurane inhalation of 0.7 MAC. Remifentanil (0.2–0.5 g·kg^−1^·min^−1^) was continuously administered to all patients, and cisatracurium was intermittently administered to maintain muscle relaxation. During the surgery, the anesthetic was adjusted according to the BIS value (controlling BIS 40–60) and hemodynamic parameters, and symptomatic treatment was used to maintain the vital signs within the normal range by administering vasoactive medications if necessary.

### Physiologic variables and index detection

Physiologic variables including HR, MAP, SpO_2_, BIS, and SctO_2_ were recorded in the three groups. Data were analyzed at six time points: induction of anesthesia (T_0_), 10 min after anesthesia induction (T_1_), 20 min after anesthesia (T_2_), 30 min after the start of surgery (T_3_), one hour after the start of surgery (T_4_), at the end of surgery (T_5_), and before leaving the post-anesthesia care unit (PACU; T_6_). SctO_2_ was the average of left and right monitoring data. The duration of surgery, anesthesia, and PACU stay was recorded.

Blood samples were collected in ethylenediaminetetraacetic acid tubes 1 day preoperatively. The levels of amyloidβ-40 (Aβ-40), Aβ-42, total tau protein (T-tau), phosphorylated tau protein (P-tau), and triggering receptors on myeloid cells-2 (TREM2) were measured by commercial enzyme-linked immunosorbent assay (ELISA) kits in accordance with the manufacturer’s instructions (BioVendor, Brno, Czechia). The absorbance was measured using VICTOR Nivo (PerkinElmer, USA) at 420 nm wavelength. Each plasma sample was double-blinded, resulting in an average concentration of two measurements.

### Neurocognitive function assessment

MoCA test was used to assess the cognitive function on the day before surgery and on the 7th postoperative day by a trained senior anesthesiologist who was blinded to the clinical information. MoCA test includes attention and concentration, executive function, memory, language, visual structure skills, abstract thinking, calculation, and orientation; it comprises a total of 11 items in eight cognitive fields, with a total score of 30, plus one point if the participant has 12 years or less of education.

The patients were grouped to calculate the standard deviation (SD) in the preoperative MoCA score, and the difference between the postoperative scores and preoperative scores was compared with the SD. If the score was reduced by ≥1 SD, it was considered that the patients had developed PND (He et al., 2019).

### Sample size and statistical analysis

The incidence of PND in elderly patients with MCI (above 65 years old) has been reported as 33.3% in non-cardiac surgery on the 7th postoperative day (Tang et al., 2014). The appropriate depth of anesthesia can reduce the incidence of PND by 22% 7 days after surgery according to the results of the pre-experimental study. With the significance set at 0.05 and the power set at 90%, the sample size required to detect differences was 60 patients. Considering that the rate of loss to follow-up is about 10%–20%, a minimum sample size of 70 patients was assigned to each group for the randomized controlled study.

Statistical analyses were performed using IBM SPSS Statistic 21.0 (SPSS Inc., Chicago, IL). The normality of the continuous variables was tested with the Shapiro–Wilk test. Differences in the continuous variables were determined by the independent *t*-test or Mann-Whitney *U* test and expressed as mean ± standard deviation, or the median (interquartile range). Categorical variables were analyzed through the *χ*^2^ test or Fisher exact test. All reported *P* values are 2-tailed. A *p* < 0.05 was considered statistically significant, and all p-values were two-tailed. A single factor linear regression analysis was performed to evaluate the correlation between the biomarkers and MoCA score. The predictive value of SctO_2_ for PND was evaluated using the receiver operating characteristic (ROC) curve and the area under the curve (AUC).

## Results

The flow diagram is shown in Figure 1. A total of 257 patients were screened for eligibility, and 224 patients were ultimately enrolled and randomized. Five patients were excluded from data analysis for the following reasons: operation time not up to standard, and a secondary operation. Nine patients were lost to follow-up. Finally, 210 patients were randomly assigned to the three groups.

### Basic characteristics and the incidence of PND

There were no statistically significant differences in gender, age, BMI, ASA classification, and years of education among the groups (Table 1). No differences in anesthesia duration, surgery duration, and PACU duration were found among the three groups. The incidence of PND in the P, S, and PS groups was 14% (10/70), 33% (23/70), and 7% (5/70), respectively, with statistically significant differences (*χ*^2^ = 16.643, *p* < 0.05).

**Table 1:** Characteristics of the patients, surgery, and anesthesia.

	**Group P**	**Group S**	**Group PS**	***P* value**
Age (years)	69.5 ± 3.3	70.1 ± 3.5	69.8 ± 3.3	0.412
Sex (Male/Female)	33/37	35/35	30/40	0.698
ASA score (II/IV)	39/31	32/38	41/29	0.280
BMI (kg/m^2^)	24.3 ± 3.8	24.2 ± 3.3	24.3 ± 3.5	0.992
Education years	6.9 ± 2.0	7.1 ± 1.8	7.3 ± 1.7	0.511
Surgery time (min)	176.5 ± 32.4	183.9 ± 36.8	183.6 ± 32.7	0.348
Anesthesia time (min)	206.7 ± 33.5	211.4 ± 36.8	207.3 ± 34.7	0.696
PACU stay time (min)	42.6 ± 8.1	43.9 ± 8.9	44.4 ± 7.7	0.420

The normally distributed data were presented as mean ± SD, the comparison was evaluated by *t*-test; categorical variables were analyzed through the *χ*^2^ test or Fisher exact test. ASA: American Society of Anesthesiologists; BMI: body mass index; PACU: post-anesthesia care unit.

### Predictive ability of preoperative plasma biomarkers for PND

**Figure 2:**
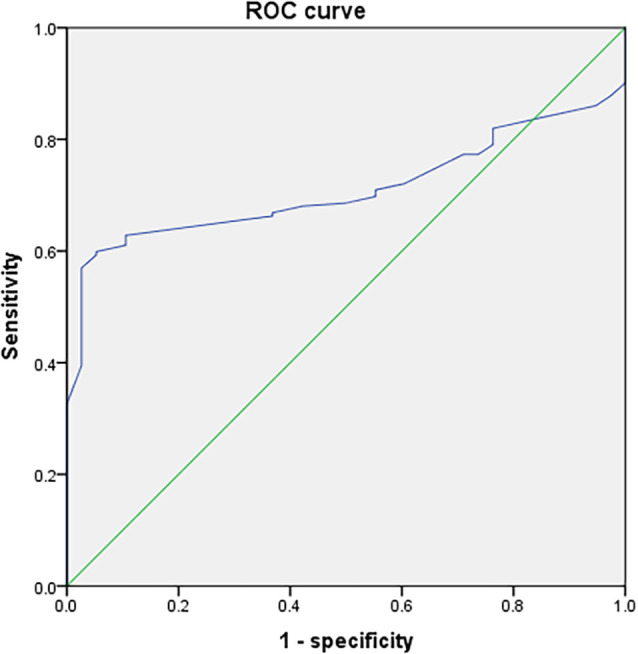
Receiver operating characteristic (ROC) curve analysis of preoperative measurement values of Aβ-42 for postoperative neurocognitive dysfunction (PND).

Univariate linear regression analysis showed that Aβ-42 had the strongest significant correlation with preoperative MoCA score (*R* = 0.697, Table 2). The preoperative value of Aβ-42 predicted PND byROC curve analysis, and the critical cutoff value of Aβ-42 predictive of PND was 28.34 pg/ml. The predicted area under the PND curve was 0.711 (95% CI, 0.644–0.777), and the sensitivity and specificity were 0.599 and 0.947, respectively (Figure 2).

**Table 2:** Correlation between P-tau, T-tau, TREM2, Aβ-42, Aβ40, and preoperative MoCA values.

	**R**	**F**	***P*value**
P-tau	−0.281	17.831	**0.000**
T-tau	−0.345	28.191	**0.000**
TREM2	−0.084	1.488	0.224
Aβ-42	0.697	196.391	**0.000**
Aβ-40	0.253	14.243	**0.000**

Bold data indicate *p* < 0.05 was considered statistically significant. P-tau, phosphorylated tau protein; T-tau, total tau protein; TREM2, triggering receptors on myeloid cells-2; Aβ-42, amyloidβ-42; Aβ-40, amyloid β-40.

### Predictive ability of Aβ-42 and SctO_2_ for PND

Based on the cognitive assessment, all patients with a preoperative Aβ-42 value below 28.34 pg/ml were divided into the PND and non-PND groups based on the MoCA score. The differences were found to be significant in age and education years between the group PND and group non-PND (*p* < 0.05). The duration of PACU stay of the group PND was longer than the group non-PND (*p* < 0.05), and the difference in surgery and anesthesia time between the groups was not statistically significant. There were no significant differences in SpO_2_, BIS, HR, and MAP between the two groups (Table 3).

**Table 3:** Characteristics of the patients, surgery, and anesthesia in the group PND and group Non-PND.

		**Group PND**	**Group Non-PND**	***P* value**
		**(*n* = 36)**	**(*n* = 69)**	
Age (years)		70.5 ± 3.4	69.9 ± 2.4	**0.007**
Sex (Male/Female)		14/22	33/36	0.382
ASA score (II/III)		20/16	32/37	0.372
BMI (kg/m^2^)		24.5 ± 3.1	24.0 ± 3.1	0.670
Education years		6.6 ± 1.5	7.4 ± 2.0	**0.045**
Surgery time (min)		180 ± 34	180 ± 35	0.959
Anesthesia time (min)		211 ± 31	204 ± 36	0.370
PACU stay time (min)		47 ± 7	42 ± 7	**0.001**
SpO_2_ (%)	T_0_	97.3 ± 1.7	97.1 ± 1.4	0.415
	T_1_	99.6 ± 1.1	99.9 ± 0.4	0.075
	T_2_	99.8 ± 0.6	99.9 ± 0.3	0.162
	T_3_	99.9 ± 0.4	99.9 ± 0.3	0.346
	T_4_	99.9 ± 0.4	99.9 ± 0.4	0.651
	T_5_	99.9 ± 0.3	99.9 ± 0.3	0.861
	T_6_	96.4 ± 1.4	96.4 ± 1.1	0.921
BIS	T_0_	95.3 ± 1.5	95.4 ± 1.7	0.706
	T_1_	48.3 ± 6.1	49.7 ± 5.0	0.229
	T_2_	49.7 ± 5.1	49.6 ± 5.1	0.924
	T_3_	49.4 ± 5.1	49.0 ± 5.1	0.712
	T_4_	50.0 ± 5.1	50.5 ± 5.0	0.683
	T_5_	62.9 ± 6.1	61.4 ± 7.0	0.291
	T_6_	92.7 ± 1.8	92.6 ± 2.2	0.765
HR	T_0_	69.4 ± 7.0	70.9 ± 6.6	0.272
	T_1_	67.4 ± 8.1	68.6 ± 8.6	0.494
	T_2_	64.8 ± 8.8	66.5 ± 7.4	0.299
	T_3_	62.5 ± 9.3	64.6 ± 7.4	0.201
	T_4_	63.4 ± 8.1	64.3 ± 7.6	0.581
	T_5_	66.1 ± 7.6	66.6 ± 8.0	0.787
	T_6_	72.7 ± 7.2	71.8 ± 7.4	0.585
MAP	T_0_	91.2 ± 7.2	90.3 ± 6.8	0.534
	T_1_	78.7 ± 7.0	81.0 ± 6.6	0.107
	T_2_	77.6 ± 7.9	79.3 ± 7.0	0.241
	T_3_	77.1 ± 7.7	80.0 ± 6.9	0.054
	T_4_	77.5 ± 6.1	79.6 ± 6.4	0.111
	T_5_	79.2 ± 7.4	81.1 ± 5.6	0.152
	T_6_	92.4 ± 10.3	92.2 ± 9.7	0.911

Bold data indicates *p* < 0.05 was considered statistically significant. PND, Postoperative neurocognitive dysfunction; ASA, American Society of Anesthesiologists; BMI, Body mass index; PACU, Postanesthesia care unit; BIS, Bispectral index; HR, Heart rate; MAP, Mean arterial pressure.

**Figure 3:**
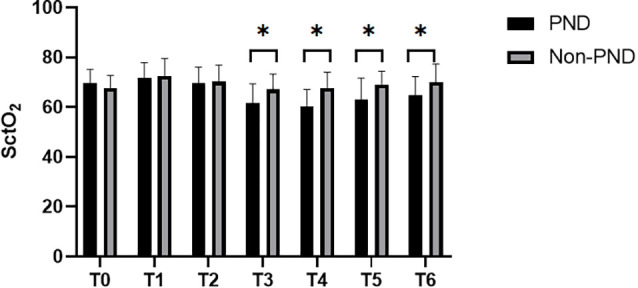
Comparison of SctO_2_ at different time points between group PND and group non-PND. *p < 0.05.

**Figure 4:**
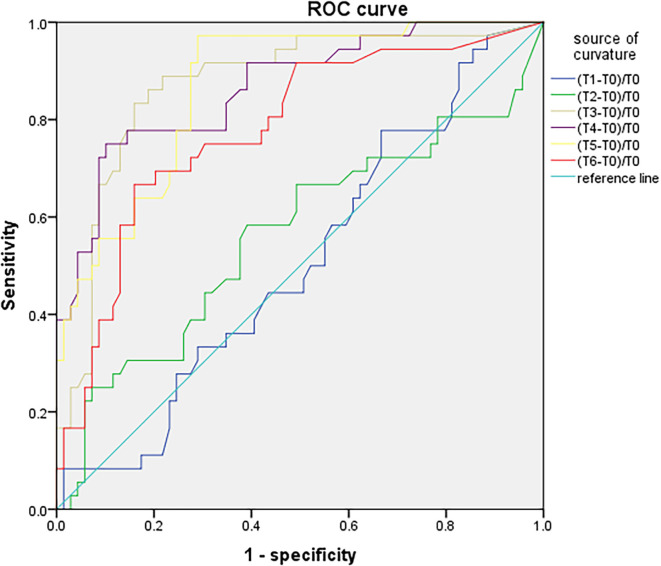
ROC curve analysis of PND predicted by the maximum percentage decline of SctO_2_ at each time point.

SctO_2_ in the group PND was lower than that in the group non-PND at time points T_3_, T_4_, T_5_, and T_6_ (Figure 3). The percentage decrease of SctO_2_ at T_3–6_ compared with the baseline value is predictive of PND. When the preoperative value of Aβ-42 was less than the critical value of 28.34 pg/ml, the cutoff value of change in SctO_2_ was determined to be 9.92% according to AUC and Jordon index. The combination predicted AUC for PND was 0.872 (95% CI: 0.797–0.947), and the sensitivity and specificity were 0.833 and 0.841 respectively (Figure 4).

**Figure 5:**
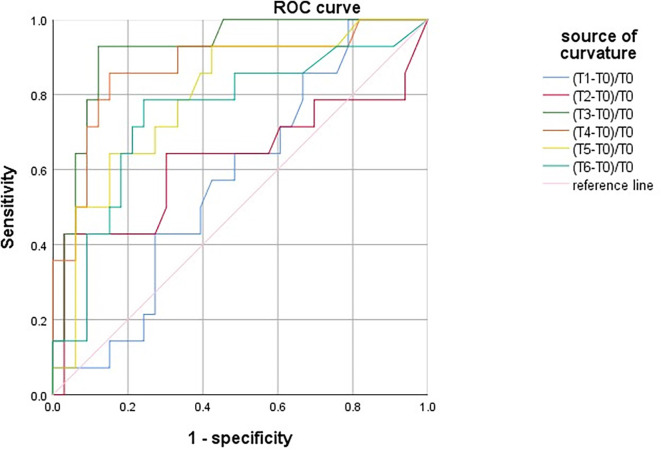
ROC curve analysis of PND predicted by the maximum percentage decline of SctO_2_ in males at each time point.

**Figure 6:**
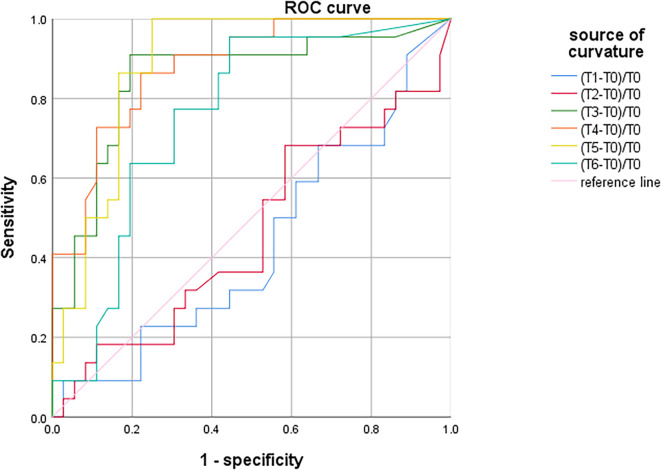
ROC curve analysis of PND predicted by the maximum percentage decline of SctO_2_ in females at each time point.

There was no significant difference in SctO_2max_% of male and female in the group PND and group non-PND (Table 4). When the preoperative Aβ-42 value was less than 28.34 pg/ml, the cutoff value of SctO_2_ change in male was 11.94% according to the AUC and Jordon index. The AUC of combined prediction of PND was 0.917 (95% CI: 0.833–1.000), and the sensitivity and specificity were 0.929 and 0.879, respectively (Figure 5). The cutoff value of SctO_2_ change in females was 6.79% according to the AUC and Jordon index. The AUC of the combined prediction of PND was 0.884 (95% CI: 0.797–0.970), and the sensitivity and specificity were 0.864 and 0.955, respectively (Figure 6).

**Table 4:** Comparison of SctO_2max_% between males and females at different time points in the group PND and group Non-PND.

**SctO_2max_%**	**Group male**	**Group female**	***P* value**
	**(*n* = 47)**	**(*n* = 58)**	
(T_1_−T_0_)/T_0_	7.50 ± 6.29	7.28 ± 7.07	0.870
(T_2_−T_0_)/T_0_	6.66 ± 5.70	7.78 ± 5.80	0.324
(T_3_−T_0_)/T_0_	8.74 ± 8.02	8.83 ± 7.69	0.951
(T_4_−T_0_)/T_0_	11.46 ± 8.07	9.88 ± 6.60	0.271
(T_5_−T_0_)/T_0_	9.43 ± 8.45	7.78 ± 6.88	0.272
(T_6_−T_0_)/T_0_	6.77 ± 5.74	7.13 ± 6.81	0.778

## Discussion

The essential finding of the prospective randomized study was that when preoperative Aβ-42 was less than 28.34 pg/ml, the intraoperative fluctuation in SctO_2_ not exceeding 9.92% could reduce the incidence of PND in elderly patients with MCI. Thus, we confirmed the predictive ability of preoperative plasma biomarkers and SctO_2_ for patients with MCI.

PND is a common complication of perioperative neurocognition in elderly patients undergoing major surgery. Spinal surgery is performed in the prone position accompany a variety of hemodynamic and respiratory alterations. Deiner and colleagues indicated that elderly spine surgery patients in the prone position were more than twice as likely to experience mild cerebral desaturation as patients in the supine position (Deiner et al., 2014). Notably, it is essential to monitor SctO_2_ in the prone spinal surgery. In our study, elderly patients undergoing spinal surgery were selected as subjects.

Sevoflurane and propofol are both commonly used anesthetics during general anesthesia. Anesthetics could theoretically also contribute to cognitive deficits by altering central cholinergic transmission through nicotinic and muscarinic receptors. The effective chamber concentration of propofol for general anesthesia is 4 μg/ml (Mahli et al., 2011), and the maintenance concentration of sevoflurane alone is 1.3 MAC (Hu et al., 2014). Studies have shown that sevoflurane anesthesia alone can aggravate PND in patients with MCI (Liu et al., 2013), while propofol with neuroprotection can improve postoperative cognitive function (Kalimefis et al., 2013). The use of compatible anesthetic drugs may reduce the dosage and the adverse reaction of a single drug. Hence, we divided the subjects into three groups for observation. Additionally, there is evidence suggesting that depth of anesthesia may be related to the risk of cognitive impairment (Radtke et al., 2013), and the intraoperative BIS value was maintained at 40–60 to eliminate the interference. In the study, the incidence of PND in the group PS significantly decreased compared with group S and group P. Thus, it is extremely necessary to optimize the anesthesia strategy for elderly patients with MCI.

The pathophysiology of PND is still unknown, although various pathophysiological mechanisms have been suggested in different situations. Preoperative cognitive impairment has been previously shown to negatively affect surgical outcomes. Adogwa found patients with preoperative cognitive impairment were at a greater than a two-fold risk of developing postoperative delirium (Adogwa et al., 2018). MCI, characterized by a transitional state on the continuum of cognitive function between normal aging and dementia, has been associated with biomarkers for AD. Identification of optimal-related biomarkers and development of monitoring strategies could enhance PND prevention and treatment practices.

Aβ has neuronal toxicity and induces neuronal apoptosis, which is a potential sign of nerve damage and persistent neuroinflammation (Wilczyńska and Waszkiewicz, 2020). Aβ-42, the fragment with 42 amino acid residues, is the main component of amyloid plaques found in the brain of patients with AD (Wilczyńska and Waszkiewicz, 2020). A second dominant isoform of Aβ is a peptide with a length of 40 amino acid residues. Neurofibrillary tangles (NFTs) formed by tau protein hyperphosphorylation and senile plaques formed by the accumulation of Aβ in the brain are the initial pathological changes in patients with AD. MCI, the early stage of AD, shows similar pathophysiological changes. Phosphorylated tau protein (P-tau) aggregates in the neurons to form NFTs, which eventually lead to neurodegeneration (Zhao et al., 2020). The elevated level of abnormal P-tau suggests the formation of NFTs in the brain parenchyma. Studies have shown that the level of P-tau protein in the CSF of patients with AD is significantly higher than that in the normal control group, and similar changes are found in patients with MCI (Ahmad et al., 2020; van Maurik et al., 2021). What’s more, high levels of plasma Aβ-42 and T-tau in MCI are associated with cognitive decline (Chen et al., 2019). Triggering receptors on myeloid cell-2 (TREM2) can stimulate the production of inflammatory cytokines during an immune response and chronic inflammation (Shi and Holtzman, 2018; Katsel and Haroutunian, 2019). Therefore, the study explored the association of these biomarkers with MCI preoperatively.

In the univariate linear regression analysis, Aβ-42 had the strongest significant correlation with the preoperative MoCA score. Studies have found a relationship between SctO_2_ and cerebral hypoxia-ischemia, resulting in cognitive decline (Mailhot et al., 2016). Optimizing SctO_2_ would potentially improve neurologic outcomes. In our study, patients were divided into the PND and non-PND groups based on the critical cutoff value of Aβ-42. SctO_2_ in the group PND was lower than that in the group non-PND at time points T_3–6_. This result was in line with that of a previous research (Colak et al., 2015), indicating that minimizing the SctO_2_ fluctuation to maintain normal cerebral oxygen supply might reduce the occurrence of PND. However, the scope of intraoperative SctO_2_ in elderly patients with MCI lacks guidance. This study enrolled geriatric patients with MCI, preoperative screening of the valuable markers and intraoperative dynamically monitoring SctO_2_ were used to determine the predictive value for PND. In this study, the maximum decrease of SctO_2_ was 9.92%, in the case of Aβ-42 less than 28.34 pg/ml. This finding provided guidance for the application of SctO_2_ to reduce PND in elderly patients with MCI.

The study demonstrated that patients in the group PND were older and had less education than the group non-PND. Consistent with previous studies, advanced age is a risk factor for PND (Scott et al., 2014), because of the decline in the central nervous system function reserve. Xu stated that the intraoperative SctO_2_ value could increase with a MAP elevation (Xu et al., 2020). In the study, there were no significant differences in MAP between the group PND and group non-PND. Thus, there are potential factors affecting SctO_2_.

With aging, the brain undergoes important structural and physiological changes. Cerebral blood flow significantly declines with age which is most prominent in the prefrontal and frontal cortex. A prospective study indicated that advanced age negatively influences baseline SctO_2_ which is otherwise influenced by sex, with women showing significantly lower values (Robu et al., 2020). Similar to previous reports, we found that males and females had similar rates of postoperative cognitive decline (Hogue et al., 2003). In addition, there was no significant difference in SctO_2max_% between males and females at different time points. Interestingly, we found that the cutoff value of change in SctO_2_ was different in gender. When the preoperative Aβ-42 value was less than 28.34 pg/ml, the cutoff value of SctO_2_ change was 11.94% and 6.79% in males and females respectively. These data suggest that, although the frequency of PND of geriatric patients with MCI is similar for males and females, females appear more likely to suffer injury to intraoperative change of SctO_2_. Gender differences in SctO_2max_% are important for the development and implementation of individualized therapeutic interventions to improve the prognosis of elderly with MCI.

MMSE scores are a widely used tool for the assessment of cognitive status in elderly subjects (Zhu et al., 2020). MoCA score is a comprehensive evaluation (including short-term memory, attention, language, time and space orientation, etc.) and can identify a wide range of changes in cognitive function with high sensitivity and specificity. Recent studies have shown that the MoCA score is the best assessment to identify MCI (Smith et al., 2007) and can sensitively identify PND (Tsoi et al., 2015). Therefore, the evaluation for PND was performed on the seventh day after surgery in this study by MoCA score.

There are limitations to the study. The sample size is relatively small, which can lead to random errors impacting the results. Due to individual and clinical scenarios differences in SctO_2_, a larger study is required to validate the relationship between the intraoperative thres hold of SctO_2_ with PND and determine the optimal SctO_2_ value that has a prognostically important relationship with PND. We only observed the cognitive function 7 days after surgery, and long-term rehabilitation was not monitored. It is recommended to evaluate all perioperative neurocognitive disorders until 12 months after surgery. The follow-up time should be extended to at least 1 year after surgery to verify less risk of group non-PND in conversion from mild cognitive impairment to AD than the group PND. Moreover, we measured the plasma levels of the biomarkers; however, the biomarkers in cerebrospinal fluid may be more representative. Nevertheless, the study has clinical implications for the prevention of PND.

## Conclusion

In summary, the randomized controlled study showed that under the conditions of preoperative Aβ-42 less than 28.34 pg/ml, the intraoperative fluctuation range of cerebral oxygen saturation should be maintained within 9.92% to reduce the occurrence of PND in geriatric patients with MCI. Preoperative plasma biomarkers along with SctO_2_ is a novel predictive strategy for the occurrence of PND in elderly patients with MCI. Biomarkers closely correlated to MCI are a predictive factor for the rapid progression of dementia. It is necessary to strengthen the monitoring of SctO_2_ undergoing surgery, especially for the elderly with preoperative cognitive decline. At the same time, multicenter studies with larger samples are needed to further accurately confirm the safety range of SctO_2_ and its relationship with PND.

## Data Availability Statement

The original contributions presented in the study are included in the article, further inquiries can be directed to the corresponding author.

## Ethics Statement

The studies involving human participants were reviewed and approved by Institutional Human Research Ethics Committee of The Third Central Hospital of Tianjin. The patients/participants provided their written informed consent to participate in this study.

## Author Contributions

YL and XX equally contributed to recruitment and initial screening of participants, data collection, and the first draft of the manuscript. HoW was responsible for data acquisition, data interpretation, and blood parameters analysis. WH contributed to study design, and revising the manuscript. XW and YW contributed to statistical analysis and revising the manuscript. PL and TZ were in charge of perioperative management, data collection, and critical manuscript revision. HaW contributed to study design, supervision, critical manuscript revision, and final approval of the version to be published. All authors contributed to the article and approved the submitted version.

## Funding

This work was supported by grants from the National Natural Science Foundation of China (82071220), Natural Science Foundation of Tianjin (20JCYBJC01290), the Science and Technology Foundation of Tianjin Health Commission (MS20013), and Tianjin Key Medical Discipline (Specialty) Construction Project (TJYXZDXK-072C).

## Abbreviations

PND: postoperative neurocognitive dysfunction
MCI: mild cognitive impairment
MoCA: Montreal Cognitive Assessment
Aβ-40: amyloidβ-40
Aβ-42: amyloidβ-42
SctO_2_: cerebral oxygen saturation
AUC: the area under the curve
AD: Alzheimer’s disease
MAC: minimum alveolar concentration
POD: postoperative delirium
PACU: post-anesthesia care unit
MMSE: Mini-Mental State Examination
NFTs: neurofibrillary tangles
TREM2: Triggering receptors on myeloid cell-2
BMI: body mass index
ASA: American Society of Anesthesiologists’ physical
CDR: Clinical Dementia Rating
ADL: activities of daily living.
